# Microspore embryogenesis: establishment of embryo identity and pattern in culture

**DOI:** 10.1007/s00497-013-0226-7

**Published:** 2013-07-14

**Authors:** Mercedes Soriano, Hui Li, Kim Boutilier

**Affiliations:** Plant Research International, P.O. Box 619, 6700 AP Wageningen, The Netherlands

**Keywords:** Microspore embryogenesis, Male gametophyte, Totipotency, Embryo patterning

## Abstract

The developmental plasticity of plants is beautifully illustrated by the competence of the immature male gametophyte to change its developmental fate from pollen to embryo development when exposed to stress treatments in culture. This process, referred to as microspore embryogenesis, is widely exploited in plant breeding, but also provides a unique system to understand totipotency and early cell fate decisions. We summarize the major concepts that have arisen from decades of cell and molecular studies on microspore embryogenesis and put these in the context of recent experiments, as well as results obtained from the study of pollen and zygotic embryo development.

## Introduction

In by far the majority of plants, embryogenesis takes place in the ovule after fusion of the female and male gametes (fertilization) and starts with the formation of the unicellular zygote. The zygote goes through species-specific cell division and histodifferentiation programs to form a morphologically complete embryo that in its simplest form comprises a shoot and a root meristem, which will produce new plant organs after germination, a hypocotyl (embryonic stem) and one or more cotyledons.

The plant kingdom is characterized by a high level of developmental plasticity, including the ability of plants to form embryos from cells other than the zygote. This phenomenon is referred to as totipotency and may be expressed as part of the normal development of some plants, as in apomixis or may be induced in tissue culture. Two major types of in vitro totipotency are observed in plants and are distinguished by the origin of the explant. Somatic embryogenesis is induced from vegetative tissues and generates plants of the same ploidy and genetic composition as the donor plant (Gaj 2001, 2004; George et al. 2008; Zimmerman 1993). Another form of totipotency is gametophytic embryogenesis, in which either male or female gametes or their associated accessory cells are induced to form embryos (Bohanec 2009; Reynolds 1997; Seguí-Simarro 2010). These cells are derived post-meiotically; therefore, the embryos that are produced in culture represent the haploid segregant progeny of the parent plant. In general, haploid embryo induction from the developing male gametophyte is more commonly applied and studied than from the female gametophyte. This is in part due to the large number of male gametophytes contained in a single anther compared to the single female gametophyte per ovule, and in part due to the ease with which anthers and pure populations of developing male gametophytes can be isolated. In this review, we focus on haploid embryogenesis from the immature male gametophyte as one form of plant totipotency. Many different terms have been used to describe this form of gametophytic embryogenesis, including androgenesis, microspore embryogenesis and pollen embryogenesis. Here, we use the more commonly used term ‘microspore embryogenesis’ to refer to the in vitro culture of the immature male gametophyte, regardless of the developmental stage of the cells that form embryos.

The haploid embryos produced through microspore embryogenesis can be germinated and grown into mature plants, but these plants are sterile due to their inability to produce gametes with a balanced chromosome number after meiosis. Chromosome doubling, which occurs either spontaneously in culture or after the application of chromosome doubling agents such as colchicine, restores the ploidy level and fertility of the derived plant (reviewed by Castillo et al. 2009).

Chromosome doubling of haploid embryos produces a plant that is homozygous at each locus in a single generation. These so-called doubled-haploid (DH) plants have been extensively exploited in plant breeding programs to increase the speed and efficiency with which homozygous lines can be obtained (reviewed in Forster et al. 2007; Germanà 2006). DH technology is traditionally used to genetically fix parental lines for F1 hybrid production, for rapid introgression of new traits through backcross conversion and to develop immortalized molecular mapping populations. DH technology is also being used to fix traits obtained through transformation and mutagenesis, to simplify genome sequencing by eliminating heterozygosity and for reverse breeding (Dirks et al. 2009; Ferrie and Möllers 2011).

The utilization of microspore embryogenesis as a biotechnology tool has been extended to a relatively diverse range of plants (Ferrie and Caswell 2011; Maluszynski et al. 2003). The ability to form haploid embryos is highly species and genotype dependent; therefore, protocols need to be developed or fine-tuned on a case-by-case basis. The decisive tissue culture parameter required to induce embryogenic growth is the application of a stress treatment, usually temperature, nutrient or osmotic stress, either alone or in combination (reviewed by Islam and Tuteja 2012; Shariatpanahi et al. 2006a). Although DH production is widely exploited, there are often one or more bottlenecks that need to be overcome before an efficient system can be established for a specific crop or genotype. The major bottlenecks in DH production are the lack or low efficiency of haploid embryo induction and the poor conversion of embryos to seedlings (Germanà 2006), and in cereals, the high frequency of albino plants (reviewed by Kumari et al. 2009; Torp and Andersen 2009). Even though the use of microspore embryogenesis has been extended to many plant families (Ferrie et al. 2011; Ferrie 2013; Seguí-Simarro et al. 2011), there are still species of agronomic (tomato and cotton) or scientific relevance (arabidopsis) that remain recalcitrant to this process.

The regenerative competence of plant cells is widely exploited at a practical level, but a deeper mechanistic understanding of the molecular basis for plant totipotency is lacking. Many studies have focused on understanding the cellular and molecular basis of microspore embryogenesis; however, the mechanism underlying this cell fate change is still largely unknown. Historically, two dicot plants (*Brassica napus* and tobacco) and two monocot plants (barley and wheat) have served as models for these studies. This review will focus on the recent advances that have been made in understanding the developmental and molecular changes that take place during microspore embryogenesis in these model systems and will use the knowledge gained from studies on other stages of plant development as a framework to better understand this process. First, we will address the commonly reported cellular changes associated with the establishment of embryo cell fate and evaluate their validity across species and culture conditions. Next, we will discuss how haploid embryos histodifferentiate; specifically, what is known about the establishment of polarity, with emphasis on the importance of exine rupture as a positional clue, and the processes that influence meristem maintenance during culture. Finally, the studies on the molecular changes during microspore embryo induction will be put in context of male gametophytic development. Overall, the current perspective on microspore embryo initiation presents a landscape in which several routes can lead to the same final destination. This intrinsic variability needs to be taken into consideration when trying to understand the basis of this developmental switch.

## Embryo fate determination in vitro

**Fig. 1:**
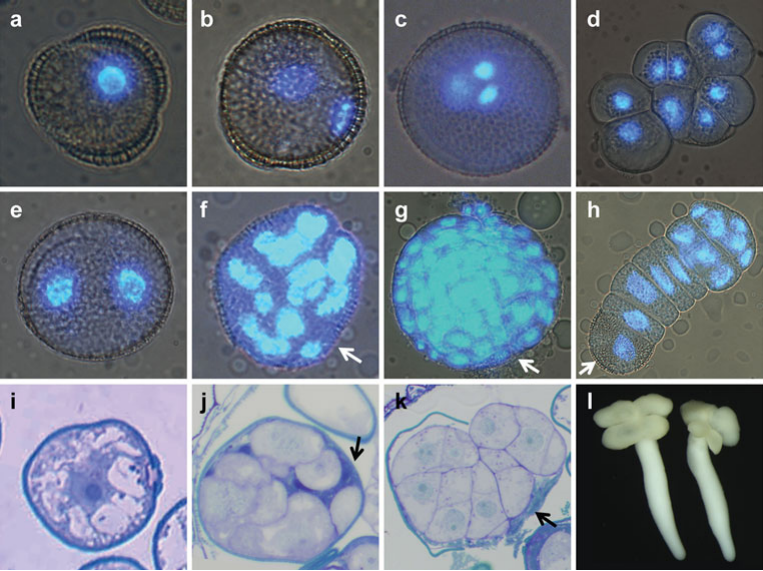
Developmental pathways observed in *B. napus* and *Triticum aestivum* microspore culture. **a**–**c** Male gametophyte development in *B. napus*. **a** Microspore; **b** binucleate pollen with a large vegetative nucleus and a smaller generative nucleus; and **c** trinucleate pollen with a vegetative nucleus and two smaller sperm nuclei. Sporophytic structures in *B. napus* (**d**–**h**, **l**) and wheat (*T. aestivum*) (**i**–**k**). **d** Callus-like structure; **e** symmetrically divided microspore with two equally sized nuclei; **f** multinucleate structure lacking clear organization that is still enclosed within the exine; **g** globular stage embryo with a well-defined protoderm; **h** suspensor-bearing embryo; **i** star-like morphology after stress treatment; **j** multicellular structure with two distinct domains; **k** multicellular structure breaking out of the exine; and **l** microspore-derived embryo at the cotyledon stage. The nuclei in a–h are stained with the nuclear dye 4′,6-diamidino-2-phenylindole (DAPI). *White arrows* indicate the localization of the exine remnants. *Black arrows* indicate the small generative-like domain in wheat

The male gametophyte or pollen grain is a two- to three-celled structure. Male gametophyte development is initiated after meiotic division of the pollen mother cell. The four products of meiosis, the microspores, each undergoes two mitotic divisions to form the mature trinucleate male gametophyte. The first mitotic division is pollen mitosis I (PMI), where the unicellular microspore (Fig. 1a) divides asymmetrically to form a large vegetative cell and a small generative cell (Fig. 1b). The vegetative cell arrests in the G1 phase (Bino et al. 1990), while the generative cell divides at pollen mitosis II (PMII) to produce a pollen grain with two sperm cells and a vegetative cell (Borg et al. 2009) (Fig. 1c). Depending on the species, PMII can take place inside the anther or during pollen germination (Reynolds 1997).

The pollen grain is a terminally differentiated structure, but can be induced to continue dividing and form haploid embryos in culture. Sunderland and Evans (1980) and Raghavan (Raghavan 1986) identified five major pathways that are thought to support embryo development. Multinucleate structures can be generated by division of the uninucleate microspore (B pathway) or in young pollen grains by division of the vegetative cell and/or generative cell (A and E pathways). In some plants, nuclear fusion between the vegetative cell and the generative cell prior to division (C pathway), as well as the initial formation of a syncytium (D pathway) have also been described. Sporophytic structures formed by division of the microspore are commonly found in *B. napus* (Zaki and Dickinson 1991), wheat (Indrianto et al. 2001), barley (Pulido et al. 2005) and tobacco (Sunderland and Wicks 1971). Sporophytic development through division of the vegetative cell accompanied by generative cell degeneration is also common (Fan et al. 1988; Reynolds 1993; Sunderland 1974; Sunderland and Wicks 1971). Reports of multicellular structures comprised of only generative-like cells are scarce, although multinucleate structures comprised of both generative-like and vegetative-like nuclei can be observed in various species including *B. napus* (Fan et al. 1988), soybean (Kaltchuk-Santos et al. 1997), wheat (Reynolds 1993, Szakács and Barnabás 1988), barley (González and Jouve 2005) and pepper (González-Melendi et al. 1996; Kim et al. 2004). These different pathways often coexist in the same cultures at varying frequencies depending on the species, the stage of male gametophyte development used as explant, and the stress treatment (Custers et al. 1994; Kasha et al. 2001). It is not clear whether all of these pathways lead to the formation of viable embryos. For example, in wheat, it was suggested that symmetric divisions (equally sized nuclei) of the immature gametophyte (Fig. 1e) would preferentially lead to embryo formation, while sporophytic structures containing both generative and vegetative-like nuclei would preferentially form callus (Szakács and Barnabás 1988). However, strong evidence to support this conclusion in this and other species is lacking, and the contribution of the different division pathways to the formation of embryos or other types of development is not known.

In most species, the stages of pollen that is most responsive for embryo induction are just before or just after PMI, although the exact window of competence is species and even genotype-specific (Bhowmik et al. 2011, Raghavan 1986). After PMII, the pollen grain enters a highly specialized transcriptional program that is different from that of both the microspore/binucleate pollen grain and other sporophytic tissues (see below) (Honys and Twell 2003). These differentiated pollen grains undergo less cell death in culture, most likely because they are more stress resistant than microspores (Thakur et al. 2010), but at the same time, they are more resistant to embryogenic induction. A compromise between a low degree of differentiation and stress resistance might be necessary to induce embryogenesis. Alternatively, the competence for embryo induction around PMI could be explained by the ability of the microspore or immature pollen grain to divide; microspores near PMI can proceed with division under stress, while younger and older stages cannot, respectively, enter or re-enter the division phase (Giménez-Abián et al. 2004; Reichheld et al. 1999). It is interesting to note that culture conditions also affect the optimal stage that is responsive to induction. For example, anther culture has recurrently been shown to require earlier stages of microspore development than isolated microspore culture (Duijs et al. 1992; Hoekstra et al. 1992). Anther tissues could provide a better environment in which microspores at early stages can develop, by providing nutrients and protection against stress. The anther wall has been proposed to isolate the microspores from the culture medium and delay the timing of induction, necessitating the use of earlier stages as starting material (Hoekstra et al. 1992; Salas et al. 2012). Lastly, microspore isolation represents an added physical stress compared to anther culture (Shariatpanahi et al. 2006b) and therefore might be more effective for late stages of pollen development that require a more intense stress treatment (Binarova et al. 1997).

Microspore embryos are formed in most species by a series of randomly oriented divisions within the surrounding exine wall. The exact point of commitment to embryo development remains unclear; therefore, the initial stages are often referred to as sporophytic growth (Fig. 1e), while multicellular, compact structures enclosed in the exine are referred to as both sporophytic structures or embryos (Fig. 1f). Upon rupture of the surrounding exine, a globular embryo is released that comprises a multicellular cluster of cells, with no evident organization and little similarity to its zygotic counterpart, with the exception of a well-defined protoderm (Fig. 1g). The formation of the protoderm is considered a marker for embryo formation (Telmer et al. 1995), and at this point, compact structures with a protoderm are normally referred to as embryos, embryoids or embryo-like structures (ELS). Eventually, these structures develop into histodifferentiated embryos that contain all the tissues and organs found in zygotic embryos produced *in planta* (Ilić-Grubor et al. 1998; Yeung et al. 1996). Most embryos are globular in shape without clear apical–basal poles and lack a suspensor structure or have a rudimentary suspensor formed by few cells. In *B. napus*, it is possible to obtain microspore embryos that show a similar, highly organized pattern of cell division as in zygotic embryos. In this pathway, a suspensor-like filament is formed by repeated transversal divisions of the microspore, followed by the formation of the embryo proper at the distal end of the suspensor (Fig. 1h). The production of this type of embryo has been optimized in *B. napus* (Joosen et al. 2007; Prem et al. 2012; Supena et al. 2008).

Not all the cultured microspores undergo sporophytic development, and of the microspores that initially switch to sporophytic growth, only a small percentage is able to form embryos. For example, in the model *B. napus* line Topas DH4079, around 40 % of the initial population divides sporophytically, while the remaining 60 % has a gametophytic identity. The final embryo yield is much lower than the initial 40 % sporophytically-divided structures (usually around 5–10 %). The majority of sporophytic structures stop growing after a few divisions and die or form callus-like structures that also eventually die (Fan et al. 1988; Telmer et al. 1995; Fig. 1d). In cereals, a high percentage of the microspores divide sporophytically, but form callus rather than embryos (Castillo et al. 2000; Fadel and Wenzel 1990; Massonneau et al. 2005; Olsen 1987).

### Changes in cellular organization

The main problem associated with defining cellular and morphological traits related to microspore embryogenesis is the heterogeneity of responses observed in culture. As mentioned above, after the stress treatment used to induce embryogenesis, many microspores arrest, divide sporophytically or continue gametophytic development. The microspores that divide sporophytically have different fates; some stop development after a few divisions, some form callus-like structures and only a small percentage form embryos. Classical cell biology studies have helped to define some of the cellular characteristics of embryogenic cells, although a direct link between cellular changes and cell fate is difficult to establish, as these studies are invariably performed on fixed material (Simmonds and Keller 1999; Zaki and Dickinson 1991). A few studies have followed the development of microspore cultures using time-lapse imaging and have provided a clearer, although often contradictory picture of the traits that characterize embryogenic microspores and the early events during embryo induction, as described below (Daghma et al. 2012; Indrianto et al. 2001; Maraschin et al. 2005c; Tang et al. 2013).

The microspores of most species are competent to form an embryo around PMI. At this stage, microspores are vacuolated and have a peripherally located nucleus. It has been proposed that one of the first effects of stress treatments on cultured microspores is the rearrangement of the cytoskeleton, with the displacement of the nucleus to the center of the cell and the formation of a preprophase band of microtubules (which is absent during normal pollen development) that marks the plane of division (Simmonds and Keller 1999; Telmer et al. 1993). The application of chemical agents, such as colchicine, cytochalasin D or n-butanol, has shown that the rearrangement of the microtubule and actin networks plays a major role in cell fate decisions, since disruption of these networks enhances or is sufficient to trigger embryo formation in the absence of a stress treatment (Gervais et al. 2000; Soriano et al. 2008; Szakács and Barnabás 1995; Zaki and Dickinson 1991; Zhao et al. 1996). These cytoskeletal rearrangements drive the displacement of the nucleus to the center of the cell, resulting in a star-like morphology in which the central nucleus is surrounded by cytoplasmic strands radiating away from the nucleus (Gervais et al. 2000). This star-like morphology has been described in several model systems and is considered the first sign of embryogenic induction (reviewed by Maraschin et al. 2005a). Live cell imaging of immobilized microspores in wheat and barley showed that a star-like morphology is associated with cell division (Indrianto et al. 2001; Maraschin et al. 2005c), but is not always a reliable marker for embryogenesis, since it can also be observed in cultured microspores that do not form embryos (Daghma et al. 2012; Maraschin et al. 2005c; Żur et al. 2013). Maraschin et al. (2005c) related embryo formation with a subpopulation of microspores in which a star-like morphology appeared later than the majority of the microspores in culture, while Daghma et al. (2012) showed that the star-like morphology can be followed by PMI and starch grain filling, which are both characteristics of pollen development.

Another cellular marker that is often associated with embryo induction is an initial symmetric division of the microspore (Fig. 1e) or the vegetative nucleus of the binucleate pollen grain. The occurrence of this type of division has been reported in a wide range of monocot and dicot species, including *B. napus* (Telmer et al. 1993, Zaki and Dickinson 1990), tobacco (Sunderland and Wicks 1971), wheat (Indrianto et al. 2001) and barley (Pulido et al. 2005) and has been correlated with the positive effect of some inducing treatments on embryogenesis, including the application of antimicrotubule agents or heat stress (Szakács and Barnabás 1995; Zaki and Dickinson 1991). An initial symmetric division is a recurrent observation in embryogenic microspore cultures; unfortunately, there is no reliable data that correlates the occurrence of a symmetric division with the embryogenic potential or embryo development, especially in cereal species (Barnabás et al. 1999; González and Jouve 2005). Recently, time-lapse imaging studies in *B. napus* showed that both symmetric and asymmetric divisions can support embryo growth, indicating that cell fate and division symmetry are not tightly coupled (Tang et al. 2013). In agreement with this, pollen that undergoes a symmetric division shows defects in the specification of the generative cell, but not a change in pollen cell fate per se (Eady et al. 1995; Tanaka and Ito 1981; Touraev et al. 1995, Twell et al. 1998).

Recently, it was shown that embryogenic structures in *B. napus* undergo autophagy and cytoplasmic remodeling (Corral-Martínez and Seguí-Simarro 2012). This massive excretion of cell material in embryogenic microspores in *B. napus*, together with the specific up-regulation of the 26S proteasome system found in barley embryogenic microspores (Maraschin et al. 2006), highlights the importance of the remodeling of cellular content as an essential first step toward elimination of gametophytic organization and progression to a new cell fate.

In general, the classical markers associated with embryogenic microspores such as a star-like morphology or an initial symmetric division cannot be considered reliable enough for early identification of the microspores that will form embryos. Moreover, the use of these morphological markers in low responding genotypes is challenging because it requires the initial screening of an enormous amount of cells (Daghma et al. 2012). Other morphological differences that have been correlated with embryogenic growth, including a thin inner layer of the pollen wall (intine) and lack of amyloplasts are difficult to confirm using light microscopy and time-lapse imaging (Maraschin et al. 2005c; Telmer et al. 1995; Zaki and Dickinson 1991). The combination of cell tracking with the use of vital stains to visualize cell viability, nuclear morphology or other cellular processes would be a valuable tool to identify early events of embryo induction. Likewise, the information generated on the molecular changes that take place in various microspore culture systems can be used as a starting point to generate reporter lines in which fluorescent reporter proteins can be tracked in real time.

### Developmental fates

In *B. napus*, haploid embryo formation is characterized by repeated randomly oriented divisions inside the exine. The multicellular cluster that develops continues dividing until the pollen wall stretches and breaks, releasing a globular structure (Fig. 1f, g). In addition to these randomly divided embryo clusters with no distinct apical and basal domains, the appearance of embryos with clear apical–basal polarity, in the form of an apical embryo proper and a distal suspensor-like structure, was occasionally reported (Hause et al. 1994; Ilić-Grubor et al. 1998; Yeung 2002). These suspensor-like structures comprise clusters of larger cells, short rudimentary filaments or uniseriate filaments attached to the root pole of the embryo. A microspore culture system was developed in *B. napus* cv. Topas DH4079 in which a high frequency of embryos bearing a suspensor structure could be obtained (Joosen et al. 2007; Supena et al. 2008). This system uses a milder and shorter stress treatment and produces a higher frequency of embryos with long uniseriate suspensors, as in zygotic embryos. These embryos are initiated by multiple transverse divisions that protrude out of the exine through an aperture or furrow and that continue dividing outside of the exine wall to form a file of cells. The distal cell divides longitudinally and produces the embryo proper (Fig. 1h).

The formation of a suspensor is important in the development of zygotic embryos to position the embryo inside of the seed, transport nutrients from the endosperm, and provide hormones to support embryo growth (Yeung and Meinke 1993). Moreover, it was shown that early patterning in microspore-derived embryos that contain a suspensor is more similar to that of zygotic embryos, pointing to a novel function of the suspensor in supporting early cellular patterning in the embryo proper. The occurrence of suspensor-bearing embryos has also been reported in microspore embryos of wheat (Rybczynski et al. 1991), but in monocots, the morphology of the suspensor in zygotic embryos is generally more unorganized than in arabidopsis and *B. napus* (Bommert and Werr 2001; Guillon et al. 2012), which could make it difficult to identify them in vitro.

The occurrence of callus-like growth often takes place side-by-side with embryo formation (Custers et al. 1999; Fan et al. 1988; Massonneau et al. 2005; Telmer et al. 1995). In tobacco, multicellular structures were described that emerge prematurely from the exine and stop growing or develop into callus (Sunderland and Wicks 1971). At least two types of disorganized sporophytic structures have been described in *B. napus.* One type of disorganized structure has a high lipid and starch content and a thick intine, and stops dividing while still inside of the exine or just after it protrudes from the exine. The other type of disorganized structure is comprised of loosely connected masses of large, multinucleate cells that eventually stop dividing (Fan et al. 1988; Telmer et al. 1995) (Fig. 1d). In maize and barley, some microspores divide to produce embryogenic calli with varying degrees of regenerability (Massonneau et al. 2005; Stirn et al. 1995). The cellular fate of these callus-like structures, whether they are initially embryogenic, gametophytic or have mixed identity, is not known. In general, it remains unclear whether callus and other cell types observed in microspore culture are formed because the initial divisions lose their embryogenic capacity, as in eggplant (Corral-Martínez and Seguí-Simarro 2012), or if these types of divisions were never embryogenic.

The two distinct forms of sporophytic development corresponding to embryo and callus formation can be differentiated in tobacco and *B. napus* microspore culture using a *35SCaMV::GUS* reporter (Custers et al. 1999). The *35S* promoter is expressed during the vegetative phase of development, but it is not active during male gametophyte development or during early embryo growth before the heart stage. Therefore, the expression of this reporter provides a means to differentiate sporophytic microspore divisions that are not committed to the embryogenic pathway. Accordingly, GUS activity driven by the *35S* promoter marked callus structures that did not develop into embryos in a low responding cultivar of *B. napus*, while it was absent in embryogenic structures. In tobacco, *35SCaMV::GUS* reporter marked an early stage of sporophytic development prior to embryo development. This suggests that the establishment of embryogenesis could take place by different developmental pathways, with a more direct switch in *B. napus* and an intermediate callus stage in tobacco.

## Polarity establishment and histodifferentiation

Embryogenic microspores show variability in their ability to undergo further growth and differentiation. The development of high-quality, histodifferentiated embryos with functional meristems is of major importance for the regeneration of DH plantlets and can be a limiting step in embryo production in some species and genotypes. The most important steps in embryo formation are (1) the establishment of apical–basal polarity, (2) the acquisition of radial polarity and formation of three main tissue layers (epidermis, cortex and endodermis) by periclinal divisions and (3) the transition to bilateral growth (with one plane of bilateral symmetry in monocots and two in dicots), characterized by outgrowth of the cotyledons (dicots) or scutellum (monocots), and the establishment of the shoot apical meristem (Bommert and Werr 2001; Sabelli 2012).

Cell division and pattern formation during zygotic embryogenesis in plants have been extensively described and studied, particularly in arabidopsis. The organization of the embryo is initially influenced by positional clues that are present prior to fertilization in the female gametophyte. In arabidopsis, the egg cell is already polarized, but briefly loses its polarization upon fertilization (Ueda et al. 2011). Subsequent changes in the organization of the cytoplasm and cell wall after fertilization (Mansfield and Briarty 1991; Mansfield et al. 1991) give rise to the zygote, which has a vacuolated polar structure (reviewed by Dodeman et al. 1997; Zhang and Laux 2011). Initially, the zygote elongates and then divides asymmetrically to form a large basal cell that will become the suspensor and the hypophysis, and a smaller apical cell that will form the embryo proper. While cell division and pattern formation in many species are a highly ordered and tightly regulated process, other species undergo less ordered division patterns with more variation in cell division planes, although a suspensor structure is always formed (Maheshwari 1950). The existence of variable division patterns suggests that cell specification is determined not only by cellular ontogeny but also by cell position, raising the question as to the importance of these controlled divisions for embryo development per se (Kaplan and Cooke 1997).

The importance of the division pattern for zygotic embryo growth is illustrated by the large number of arabidopsis mutants that show altered cell division during early embryogenesis leading to defects in embryo formation. For example, *knolle* mutants, which lack an epidermal cell layer, cannot grow into a normal embryo and are defective in the establishment of the apical–basal axis (Mayer et al. 1991). Both *fass* and *fackel* mutants are unable to orient their division planes. However, while the *fackel* mutant shows mislocalization of the meristems and is seriously compromised in embryo development (Schrick et al. 2000), in the *fass* mutant, the distinct cell identities are correctly established, although they cannot be identified morphologically. These observations suggest that in some cases, an ordered series of cell division is not required for differentiation (Torres-Ruiz and Jurgens 1994). In maize, seven out of ten mutants defective in the first asymmetric division of the zygote failed to develop an embryo proper (Sheridan and Clark 1993). Therefore, even in monocots species, where embryo divisions are not as tightly ordered as in arabidopsis, early embryo patterning during seed development can be decisive for later embryo development.

The initial morphology of in vitro cultured embryos, whether derived from somatic or gametophytic tissue, is generally much less organized than their zygotic counterparts (Mordhorst et al. 1997; Yeung et al. 1996). The initial embryonic divisions of microspore embryos are random and produce a cluster of cells in which different cell types cannot be readily distinguished (Fan et al. 1988; Telmer et al. 1995; Yeung et al. 1996). A suspensor is generally not formed. The development of the globular structure begins to mimic that of zygotic embryos once the embryos break out of the exine and is marked by the establishment of a protoderm layer (Telmer et al. 1995). In maize, the epidermal marker *LTP2* was specifically expressed in embryo forming structures and not in callus (Massonneau et al. 2005). The formation of a protodermal layer is followed by the enlargement of the apical region and by a transition stage in which the cotyledons (or the scutellum) start to form (Ilić-Grubor et al. 1998; Maraschin et al. 2003; Yeung et al. 1996). It is not clear how apical–basal polarity is established in microspore embryos, i.e., whether it is established in the microspore, during the first sporophytic divisions inside of the exine, or later in development. In somatic embryos, the surrounding tissues (when present) can provide positional clues, but polarity can also be established in the absence of such tissue. Also, gradients of exogenously applied plant hormones can be established and direct embryo growth and division (Friml et al. 2003). Microspore embryos can develop in the absence of external hormones and sporophytic tissues. The question then arises as to how these unorganized structures form a complete embryo in the absence of an initial formative division and without a supporting suspensor or external positional clues.

### Preexisting polarity cues

In contrast to zygotic embryos, the first embryogenic division in microspore culture is often symmetric (Simmonds and Keller 1999; Zhang and Laux 2011). It was proposed by Hause et al. (1993) that an initial asymmetric cell division was not required in microspore embryogenesis because of the high degree of polarization that is already present in the microspore. In cereals, microspores are polarized due to the presence of a single round aperture in the pollen wall. In agreement with this observation, in cereals, early embryogenic multicellular structures contained within the exine are often characterized by two heterogeneous cell domains; a smaller domain comprised of small, dense cells and a larger domain comprised of larger cells (Bonet and Olmedilla 2000; Dubas et al. 2010; Góralski et al. 2005; Magnard et al. 2000; Maraschin et al. 2005b; Testillano et al. 2002). In maize, the large domain shows similarity to endosperm, including a coenocytic organization with incomplete cell walls, synchronous cell division, vacuolated cytoplasm and starch granules (Testillano et al. 2002). Endosperm-specific gene expression was detected in these structures, but was not restricted to the endosperm-like domains (Massonneau et al. 2005). The microspores of dicots like *B. napus* also show polarized development, with a central vacuole and the nucleus localized to the periphery. However, unlike cereals, embryogenic structures in *B. napus* are usually uniform clusters of cells in which no distinct domains can be distinguished (Fan et al. 1988; Joosen et al. 2007).

The formation of suspensors in *B. napus* could arise due to preexisting polarity factors in the microspore that remain after exposure to a mild stress (Supena et al. 2008). In microspores subjected to a longer and stronger heat stress, polarity clues from the microspore would be erased and result in symmetric division of the microspore and the formation of randomly divided structures. It was also suggested by Straatman and Schel (2001) that suspensor-like structures result from aberrant growth induced by the early rupture of the microspore exine wall. Interestingly, recent work by Tang et al. (2013) suggests that the partial breakage of the exine increases the formation of suspensor-bearing embryos. Therefore, it would be reasonable to think that polarity clues derived from specific characteristics of the microspores (i.e., cell wall properties, remnants of cellular organization), and/or by the early rupture of the pollen wall, could trigger the formation of polarized suspensor structures.

### Exine rupture

Exine rupture is an important step in microspore embryo growth. Most of the sporophytic divisions that fail to form an embryo stop dividing before the exine ruptures (Maraschin et al. 2005b) or when it breaks prematurely (Sunderland and Wicks 1971; Telmer et al. 1993). Several reports have shown that the site of rupture plays an important role in polarity establishment. Regardless of the species, exine remnants often remain attached to the root pole, suggesting that the apical domain of the embryo coincides with the site of exine rupture (Hause et al. 1993; Ilić-Grubor et al. 1998; Indrianto et al. 2001; Tang et al. 2013). In *B. napus*, the male gametophyte contains three pollen apertures and two types of exine rupture during microspore culture have been described; type I in which the cells grow and increase volume, protruding out of the apertures and type II in which the structure grows in a uniform way producing the even stretch of the exine (Nitta et al. 1997). It is not known whether preexisting polar growth drives the site of exine rupture or if polarity is established as a consequence of the differential rupture. The first morphological sign of polarity establishment in *B. napus* is the disappearance of starch granules at the site of exine rupture, which will become the future apical pole (Hause et al. 1994). Studies in brown algae (*Fucus*) embryos show that the cell wall provides positional information to establish a polar axis and orient the first cell division plane of the zygote and that differences in cell wall composition are important for cell fate determination (Belanger and Quatrano 2000). Localized vesicular secretion is essential for remodeling of the cell wall and for the establishment of polarization in *Fucus* and has also been shown to be important in vascular plants for polar transport of the morphogen hormone auxin (Belanger and Quatrano 2000; Geldner et al. 2003). The cell wall changes that characterize the switch to microspore embryogenesis, include the moderate growth of the innermost pecto-cellulosic wall and intine (Bonet and Olmedilla 2000; Schulze and Pauls 2002; Solís et al. 2008; Telmer et al. 1995), an increase in pectin esterification (Bárány et al. 2010) and the differential localization of arabinogalactan epitopes (El-Tantawy et al. 2013). These or other changes in the cell wall properties could be important for the ability of induced microspores to develop into embryos and require further study. The role of the cell wall and other structural cell components in the regulation of plant growth is receiving increasing attention, especially in light of their importance as mediators of mechano-stress signaling and the regulation of organ growth (Braybrook and Peaucelle 2013 and references therein).

Premature exine rupture seems to be detrimental to further embryo growth. However, in *B. napus* suspensor-bearing embryos, the exine ruptures after only a few cell divisions, but the suspensor filament still develops and is thought to emerge through one of the pollen apertures. Microspores with a partially broken pollen wall, so-called exine-dehisced microspores (EDM) can be obtained by breaking the exine by physical stress. The EDM elongates and protrudes of out of the ruptured site and often gives rise to the formation of well-developed suspensor embryos (Tang et al. 2013). The orientation of the first division plane in these EDM is predominantly transversal to the axis marked by exine rupture (which is defined by the remnants of the exine in one extreme of the cell), and therefore, it has been proposed that the location of exine rupture determines the division plane via mechanical stress. This work, together with the observations of Hause et al. (1994), shows that in *B. napus*, the site of exine rupture can direct the polarity axis of the embryo and points to a role for the pollen wall in microspore embryo organization.

In barley microspores, which have only one aperture, the embryo consistently breaks out of the exine at the side opposite to the aperature. This process has been proposed to be regulated by cell death of the small cell domain, which is localized at the site of rupture (Maraschin et al. 2005b). In barley, the small cell domain has been associated with repeated division of the generative cell and its presence is important to promote exine rupture: homogeneous multicellular structures that lack this domain fail to break the exine and do not develop further (Maraschin et al. 2005b). The question that remains is how this cell death process is regulated, i.e., whether it is the cause or consequence of exine rupture. It would be interesting to determine whether PCD is regulated in systems were morphological distinct domains might be absent prior to pollen wall rupture, such as *B. napus*. It is clear that in some cases, the establishment of polarity precedes rupture of the microspore wall and determines both the site of the rupture and the orientation of the body axis of the embryo. However, there is increasing evidence for the role of the pollen wall in defining the apical–basal axis. The variability that seems to operate in different species should be explored to gain insight into the pathways that lead to plant and embryo polarity and self-organization.

### Maintenance of meristem integrity

Once the exine breaks, the main tissue layers of the embryo are formed, which include the protoderm, the procambium and the ground tissue layers that will form, respectively, the epidermis, the vascular tissue and the parenchyma. The apical-basal axis of the embryo is established by the formation of the meristems. Although embryos produced in vitro initially develop well-formed meristems, these meristems may degenerate later in culture (Stasolla et al. 2008). Embryos that contain degenerated meristems cannot be converted directly into plants. This degeneration primarily affects the shoot apical meristem (SAM) and is characterized by acquisition of parenchymous features such as the formation of intercellular spaces and vacuolation, as well as loss of meristem identity (Belmonte et al. 2005). Belmonte et al. (2005, 2006) proposed that the degeneration of the meristems during in vitro culture was due to the requirement for a more oxidized environment during late embryo development. In agreement with this hypothesis, abnormalities in SAM organization that are observed in the late phases of microspore embryo development can be rescued by a lower cellular redox state, obtained by chemical inhibition of de novo glutathione synthesis (by application of buthionine sulfoximine, BSO) or by treatment with the oxidized form of glutathione (Belmonte et al. 2006, 2011). Glutathione and ascorbate are molecules with both oxidized and reduced forms that play a role in the detoxification and scavenging of reactive oxygen species and in the regulation of the redox cellular state. BSO treatment affects ascorbate metabolism, producing lower ascorbate levels in treated embryos, and activated expression of meristem-specific genes including *ZWILLE*, *SHOOTMERISTEMLESS* and *ARGONAUTE 1* (Stasolla et al. 2008). The more oxidized environment produced by BSO also reduces the level of ethylene and induces gene expression associated with the embryo maturation phase of zygotic embryo development, including ABA response proteins and late-embryogenic abundant (LEA) proteins. Overall, the change in redox status during embryo development produces a metabolic switch needed for the embryos to reach maturity. This change is proposed to be mediated by an ABA response, since ABA treatment produces similar effects on embryo maturation and conversion frequencies (Belmonte et al. 2006; Ramesar-Fortner and Yeung 2006).

Enhancement of proper SAM functionality in microspore embryos was also attained by the overexpression of *SHOOT MERISTEMLESS* (*STM*), a Class I knotted-like homeodomain transcription factor that functions in SAM initiation and maintenance (Barton and Poethig 1993). *STM* overexpression maintains expression of cell cycle machinery genes and characteristics of meristematic cells, while repressing the cell wall modifications typical of cell differentiation (Elhiti et al. 2012). Overexpression of *STM* induced expression of known embryogenesis regulatory genes and also reduced reactive oxygen species (ROS) by the increase in scavenging enzyme activity and by increased ascorbic acid (Elhiti et al. 2012). Elhiti et al. proposed that STM delays cellular differentiation through a decrease in ROS levels and by reducing cell wall rigidity.

It has been proposed that the maintenance of cellular brassinosteroid levels is required for the formation of functional apical meristems. This view is supported by the increase in the number and quality of microspore-derived embryos upon treatment with externally applied brassinolide, whereas treatment with brassinazole, a brassinosteroid biosynthetic inhibitor, has the opposite effect (Belmonte et al. 2011). Interestingly, upon brassinazole treatment, the ascorbate and glutathione pools in microspore embryos switch toward an oxidized state, supporting a role of brassinosteroids in the regulation of the redox state during embryo development. A role for brassinosteroids in control of the cellular redox state of the SAM during the transition to the maturation phase of development in zygotic embryos has not been described.

## Molecular control of haploid embryo induction

The developmental starting point for microspore embryogenesis is the male gametophyte. Therefore, to understand the molecular basis for haploid embryo induction, this change in development must be placed in the context of the normal pathway of pollen development. This comparison is especially important, when one considers that the vast majority of cultured microspores and pollen do not form embryos, but rather continue gametophyte development or arrest and die.

The developmental stage of the immature male gametophyte is a critical factor that influences its embryogenic potential. Transcriptome analyses in arabidopsis (Honys and Twell 2004) and wheat (Tran et al. 2013) have shown that the transcriptomes of microspores and bicellular pollen are highly similar. Their transcriptomes show little overlap with that of mature pollen, but rather are more similar to those of other sporophytic stages of plant development (Honys and Twell 2004; Joosen et al. 2007; Tran et al. 2013; Whittle et al. 2010). The microspore transcriptome is characterized by a higher proportion of transcripts encoding structural proteins, as well as proteins involved in translation and metabolism (Whittle et al. 2010). As pollen matures, there is a shift toward expression of fewer, but more highly abundant, pollen-specific transcripts that mainly encode proteins involved in pollen germination and tube growth (Becker et al. 2003, Loraine et al. 2013). The course of male gametophyte development is therefore characterized by a shift toward a higher degree of specialization. The initial similarity between the microspore/bicellular pollen and sporophytic stages of plant development may provide the developmental competence that is needed to switch from gametophytic to sporophytic growth during microspore embryogenesis (Whittle et al. 2010).

Gene expression studies aimed at understanding the molecular basis of microspore embryogenesis have relied on comparison between cultures induced to undergo embryogenesis and non-induced cultures containing developing pollen. Although these studies have a common goal, it is difficult to develop a common picture of the molecular changes that accompany the switch from pollen development to haploid embryogenesis. Firstly, the available studies are focused on different model species, each induced with one or more treatments, including high temperature stress, nutrient starvation and/or osmotic stress and each with different starting material, e.g., isolated microspores or anthers. Secondly, each of these studies has been performed using different, mainly low-throughput, approaches to identify transcripts of interest, including screening of cDNA libraries (Hosp et al. 2007), sequencing of expressed sequence tags (ESTs, Malik et al. 2007; Tsuwamoto et al. 2007), targeted expression analysis of candidate genes (Sánchez-Díaz et al. 2013) and custom (Joosen et al. 2007; Maraschin et al. 2006) and commercial (Muñoz-Amatriaín et al. 2006) DNA arrays. A third problem is the low embryogenic response of the cultures, although approaches to enrich for embryogenic microspores (Maraschin et al. 2006) or specific sequences (Malik et al. 2007) have been carried out. Given the limitations outlined above, we discuss the major concepts that have emerged from these studies.

### Deregulation of pollen development

It is generally assumed that microspore re-programming to embryogenesis is achieved, in part, by repressing gametophytic development. In barley, microspore embryogenesis is induced by exposing cultured anthers to starvation and to osmotic stress using mannitol. A highly embryogenic fraction of microspores can be purified by density centrifugation after 4 days of culture in the anther (Maraschin et al. 2005c). Comparison of the gene expression profiles of this enriched fraction with pollen showed that while pollen development was characterized by the expression of starch biosynthesis genes, the embryogenic microspore fraction showed the opposite trend: a decrease in the expression of starch biosynthesis genes and an increase in expression of genes involved in sugar and starch hydrolysis (Maraschin et al. 2006). This observation is in agreement with many studies showing that starch accumulates during late pollen development and that its accumulation in microspore culture is detrimental to embryo progression (McCormick 1993). This data, although limited, also imply that at least some genes involved in pollen development are down-regulated during embryo induction (Maraschin et al. 2006).

In *B. napus*, isolated microspores develop into embryos after exposure to heat stress. The first sporophytic divisions are observed after 1–2 days of culture, and by 5 days of culture, globular-shaped multicellular structures are formed that begin to emerge from the surrounding exine. Initially, the vast majority of cells in culture follow the gametophytic pathway, but around 5–6 days of culture, the pollen grains burst open and die. Microarray analysis has shown that 2 day-old heat-stressed microspore cultures are highly similar to pollen cultures (Joosen et al. 2007). Only at 5 days of culture, when the pollen is dead, could genes that are differentially expressed between pollen and embryogenic microspore cultures be identified by microarray analysis. It was not clear from this analyses whether the gene expression profiles associated with embryogenic microspores at 2 days of culture were swamped by the highly abundant, stable, late pollen transcripts, or whether both pollen and embryo development coincided in the same cell types. It is possible to identify proteins that are differentially expressed in 2-day-old induced cultures compared to pollen. Although co-existence of pollen and embryo identities in the same structure cannot be excluded, differential protein expression as early as 2 days of culture does support the observation that the abundance of late pollen transcripts in the RNA samples is due to the presence of non-translated pollen mRNAs (Mascarenhas 1990, 1993; Ylstra and McCormick 1999) and that proteomics may provide a more sensitive approach to identifying totipotency-related pathways. Suspensor-bearing embryos develop much slower than suspensor-less embryos. After 8 days of culture, embryos with a long suspensor and a one- to two-celled embryo proper have developed, while the gametophytic cells are no longer viable. Microarray analysis of these samples shows clearly different expression profiles from those of developing pollen, indicating that, at least in this pathway, embryogenic and gametophytic gene expression do not co-exist in the same structures. Malik et al. (2007) showed using hand isolated 5 day-old embryos that lack suspensors, that both pollen and embryo markers are expressed in the same samples. In support of this, Pulido et al. (2009) have shown that the late pollen gene *PG1* is expressed in few-celled sporophytic structures found in barley cultures, but disappears as sporophytic growth progresses. The question of whether and to what extent active pollen and embryo gene expression occur in parallel in microspore embryos is intriguing, but cannot be answered at this time. Live imaging using rapidly turned-over pollen reporters together with embryo identity reporters will be needed to determine whether pollen genes are actively expressed in embryogenic microspores or whether these mRNAs are simply remnants of highly stable transcripts.

### Establishment of embryo identity

As mentioned above, studies aimed at identifying the early molecular events that accompany haploid embryo induction have been hampered by the presence of highly abundant pollen transcripts. A few studies have identified differentially expressed sequences using methods to subtract pollen-expressed genes (Joosen et al. 2007; Malik et al. 2007; Tsuwamoto et al. 2007), but these analyses were performed late in the development of the culture, when sporophytic clusters are already present. The study by Maraschin et al. (2006) is to our knowledge the only study that examined gene expression in microspores that were induced to undergo embryogenesis, but had not yet divided. When the expression profile of these cells was compared to microspores and developing pollen, they found evidence for a role for proteolysis, stress response, inhibition of programmed cell death and signaling pathways in embryo induction that could be separated from effects of the stress treatment used to induce embryogenesis. Unfortunately, the number of genes examined in this study is small, precluding a more global analysis of these pathways activated during haploid embryo induction.

Two other studies in *B. napus* examined gene expression profiles in embryos at a slightly later stage of development, starting from 2 to 3 days of culture, when induced microspores had already gone through a few sporophytic cell divisions (Joosen et al. 2007; Malik et al. 2007). Malik et al. (2007) noted a sharp decrease in the expression of protein synthesis machinery genes at day 3 of microspore culture and associated this drop in expression with a switch to the embryogenic pathway. The observation may be explained by the dominance of late pollen-expressed genes at this stage and the normal decrease in expression of protein machinery genes during late pollen development (Honys and Twell 2004; Joosen et al. 2007; Whittle et al. 2010). In support of this, expression of protein synthesis machinery genes increased in 5- and 7-day-old cultures (Joosen et al. 2007; Malik et al. 2007), coinciding with the loss of pollen and increase in sporophytic growth. This point highlights the difficulty of analyzing gene expression profiles in highly heterogeneous cultures in which many different developmental pathways co-exist.

**Fig. 2:**
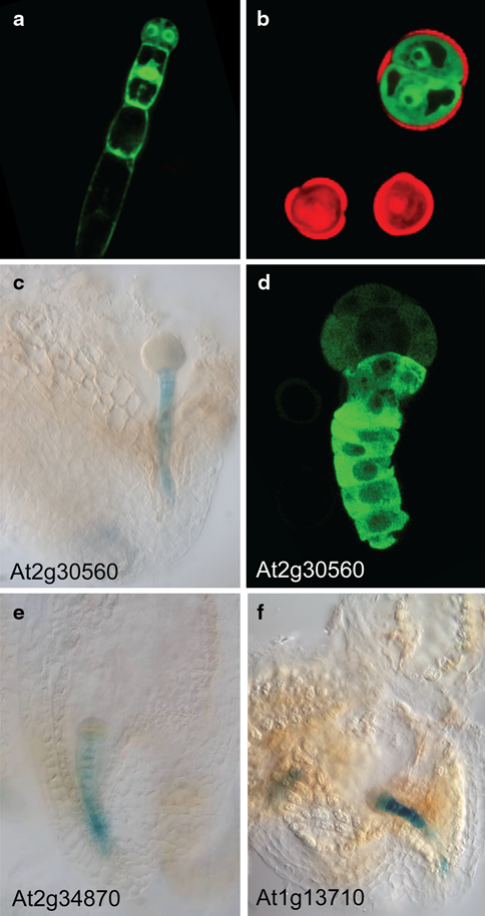
Expression of suspensor and embryo markers identified in *B. napus* microspore culture. Expression of an arabidopsis *LEC1::LEC1:GFP* reporter in **a** the two-celled embryo proper and suspensor of a *B. napus* zygotic embryo and **b** a sporophytically-divided microspore in *B. napus*. GFP expression in **a**, **b** is shown in *green* and autofluorescence in **b** is shown in *red*. The two smaller microspores in **b** do not show GFP expression. Expression of arabidopsis orthologs of *B. napus* suspensor-expressed genes in arabidopsis zygotic embryos (**c**, **e**, and **f**) and a *B. napus* microspore embryo (**d**). The *lines* shown in **c**, **e**, and **f** are promoter: GUS reporters and the *line* shown in **e** is a promoter: GFP reporter. The corresponding arabidopsis gene identifier for each reporter is indicated

While detailed studies on the events prior to embryogenic division are lacking, there is much more known about the expression of early embryo genes in microspore culture, specifically in *B. napus* (Joosen et al. 2007; Malik et al. 2007; Tsuwamoto et al. 2007). Malik et al. (2007) identified a large number *B. napus* ESTs that show strong sequence similarity to known arabidopsis embryo-expressed genes, in particular transcription factor genes. The expression of 24 of these genes was rigorously examined using quantitative RT-PCR in induced and non-induced microspore cultures, during seed development and during other stages of sporophytic development. Based on these results, they were able to identify a set of genes that are expressed in haploid and zygotic embryo development, but not during pollen development. These genes include *FUSCA3*, *LEAFY COTYLEDON1 (LEC1)*, *LEC2*, *BABY BOOM (BBM)*, two WUSCHEL-related homeobox (WOX) genes, *WOX2* and *WOX9*, and *ABSCISIC ACID INSENSITIVE3*. Although ESTs for these genes were only detected after 7 days of culture, their expression could be detected by RT-PCR much earlier, at 1–2 days of culture, suggesting that embryo cell identity is established as early as the first few sporophytic cell divisions. The utility of a subset of these genes as early markers for embryogenic growth in genotypes differing in their ability to form haploid embryos was also examined. Only the expression of one of the markers, *LEC2*, could distinguish between embryogenic and non-embryogenic cultures at 3 days, but all of them distinguish the same cultures at 7 days. The low correlation with embryo formation is not surprising, as the expression of the marker depends on many factors, including its own expression level, the proportion of embryogenic cells in the culture, and whether the genotype is negatively affected in a pathway in which the marker gene normally functions. Unpublished work from our laboratory suggests that a LEC1:GFP fusion, that is specifically expressed in embryos during seed development (Fig. 2a), marks embryogenic microspores in culture in a poorly responding genotype as early as 3 days after the start of culture (Fig. 2b).

The *B. napus* suspensor-embryo system also proved to be a valuable tool to identify early embryo-expressed genes (Joosen et al. 2007). Suspensor-bearing embryos develop more slowly than conventional cultures so that by the time the embryo proper has reached the two-cell stage the pollen that co-develops in the culture has already died. Comparison of conventional embryos without a suspensor and embryos with a suspensor (few-celled to globular stage embryo proper) identified a set of 43 genes whose expression is significantly up-regulated in embryogenic microspore cultures compared to the male gametophyte. The suspensor expression of a number of these genes has been confirmed in arabidopsis and *B. napus* (Fig. 2c–f). This model system for in vitro suspensor production offers a novel tool for the isolation and molecular characterization of this poorly accessible tissue.

Based on the above, we can conclude that the molecular activation of the embryo pathway is an early event in haploid embryo induction, at least in *B. napus*. Other studies in barley and tobacco did not specifically describe expression of early embryo-expressed genes in microspore culture (Hosp et al. 2007; Maraschin et al. 2006; Muñoz-Amatriaín et al. 2006). Nonetheless, it is still not clear which gene expression events, if any, precede the activation of embryo gene expression in microspore embryo induction. Ectopic expression of the *LEC1*, *LEC2* and *BBM* transcription factors in seedlings is sufficient to induce activation of embryo-expression programs, as well as the de novo induction of somatic embryo formation (Boutilier et al. 2002; Lotan et al. 1998; Stone et al. 2001). Given the sporophyte-like identity of the microspore and bicellular pollen grain, de novo expression of these transcription factors in response to stress could be sufficient to induce a switch to totipotent growth. On the other hand, the expression of embryo markers may simply represent an early, but secondary event that is set in motion by the stress treatment.

## Conclusion and perspective

Microspore embryogenesis has been extensively studied, but still the mechanism that drives this process, from the initial embryogenic cell divisions to the formation of histodifferentiated embryos, is not understood. Many of the early cell biological observations on microspore embryo induction are now being revised or even discarded in light of live imaging studies. The picture is even less clear at the molecular level, where different starting materials, type and duration of induction treatment, and gene expression platforms have been used to probe the embryogenic microspore. To proceed further requires a collaborative approach in which live imaging is combined with cell and molecular analyses. The different culture systems need to be stripped down to their simplest elements to facilitate a direct comparison, and high-throughput DNA and protein sequencing techniques are needed to identify and compare transcripts in microspores and pollen, as well as in embryogenic and stressed, non-embryogenic microspores. The identified genes need to be definitively linked to microspore embryogenesis pathway, rather than stress response, using genetic and genomics approaches, such as mutant analysis, as well as time-lapse imaging of candidate and other pathway markers in live cells.
